# Fighting for care: how can we better support people with multiple long-term conditions who are accessing community mental health groups? A qualitative interview study within a UK arts therapies trial

**DOI:** 10.1136/bmjopen-2025-103035

**Published:** 2026-03-03

**Authors:** Lauren Hounsell, Emma Millard, Emma Medlicott, Emma Fry, Jane Fernandes, Catherine Carr

**Affiliations:** 1 Unit for Social and Community Psychiatry, East London NHS Foundation Trust, London, UK; 2 Unit for Social and Community Psychiatry, Centre for Psychiatry and Mental Health, Wolfson Institute of Population Health, Queen Mary University of London, London, UK; 3 Oxleas NHS Foundation Trust, London, UK; 4 East London NHS Foundation Trust, London, UK

**Keywords:** Multiple long-term conditions (MLTCs), mental health, arts therapies, community groups, qualitative interviews, group therapy

## Abstract

**Objective:**

To explore the impact of multiple long-term conditions (MTLCs) and a comorbid mental health condition on decision-making processes, attendance and engagement in NHS community-based therapy groups.

**Design:**

Qualitative in-depth interviews analysed using reflexive codebook analysis as part of a study within a trial.

**Setting:**

Secondary community mental health teams from two UK sites.

**Participants:**

Purposive sample of 20 participants recruited to a randomised controlled trial of group therapies (arts therapies and counselling) holding a mental health diagnosis and self-reported as having at least one additional physical health condition.

**Results:**

Six themes were constructed: (1) MLTCs influenced arts modality choices and goals; (2) importance of planning ahead to be organised; (3) the journey loomed over participants; (4) the impact of MLTCs on group attendance and participation; (5) the group was valued and important; (6) determination and fighting to get what I need.

Decisions about arts modalities and group attendance were based on a self-perceived level of felt capability. It was important for participants to plan in advance and feel informed ahead of making commitments, enabling them to prepare and manage symptoms. Travelling to the groups was dreaded, and many participants required support with travel in order to attend. Managing symptoms during the journey and groups was challenging; however, participants had a strong determination to uphold the commitment to attend despite their difficulties, as the group was highly valued.

**Conclusions:**

MLTCs have a large impact on people’s capacity to engage in community groups, requiring additional planning and effort. The scale of this impact is often not recognised. Despite this, the benefits of groups for people with MLTCs are especially important, including motivation to leave the house, opportunities for socialisation and a means of reaching one’s own goals. Clinicians are recommended to accommodate the needs of MLTCs when designing community group interventions and consider multiple attendees with MLTCs in the group composition to improve attendance and group engagement.

**Trial registration number:**

ISRCTN88805048.

**STRENGTHS AND LIMITATIONS OF THIS STUDY**

- This qualitative interview study was embedded in a large randomised controlled trial of group arts therapies, with an active control of group counselling.
- Sampling of participants ensured representation of all types of group therapies delivered in the trial, of high, low and non-attenders, and with a range of mental health diagnoses.
- Participants may have previously been recruited into the study, or completed follow-up assessments with their interviewer, which may have aided rapport or influenced what participants felt able to share.
- Participants were of an older age (43–77), which was representative of the ages of *E*ffectiveness of group arts therapies compared with group counselling in diagnostically heterogeneous patients: *Ra*ndomised controlled trial study participants; however, we may have missed the experiences of younger participants with multiple long-term conditions.
- Musculoskeletal conditions were most reported and may have driven themes relating to medication and pain management.

## Background

 The increased prevalence of multiple long-term conditions (MLTCs) among people with a mental health condition compared with the general population in the UK is well established^1 2^ with rates continuing to rise.^3^ The term MLTC refers to the existence of two or more long-term conditions in an individual^4^ and the increasing occurrence among the population has been regarded as one of the leading challenges facing health services in the UK.^5^ Life expectancy reduces by 10–20 years for people with mental health conditions,^6 7^ and preventable physical conditions such as cardiovascular diseases are among the leading causes of mortality among this group.^8^ Having MLTCs significantly increases the complexity of care required,^9^ including attending multiple health services to treat conditions individually. A recent population study uncovered that 14.8% of the population in England are living with MLTCs, with the most common physical conditions in adults being hypertension, osteoarthritis, asthma and cardio-metabolic factors (diabetes, coronary heart disease).^10^ It is becoming increasingly imperative for health services to adapt to ease this burden and fulfil the growing care needs of the population.^5^ However, current clinical guidelines do not reflect the experiences and needs of people with MLTCs,^3^ which requires attention.

Group therapies have been found to provide beneficial outcomes for those presenting with mental health conditions, such as improving psychiatric symptoms, fostering peer support and equipping people with skills to better manage symptoms.^11^ Attendance of community interventions, such as group therapies, can often be low for mental health populations,^12^ with limited accessibility and insufficient information ahead of the group found as some of the barriers to attending.^12^ Recommendations from this research suggested that increasing participants’ autonomy and enabling more choice could improve their engagement. Furthermore, a meta-analysis found that accounting for mental health patients’ preferences in psychosocial treatments was integral to improving engagement.^13^ These studies show that being given a say in treatment is important for people and has an impact on attendance and engagement in therapeutic interventions. However, there may be different factors at play when having both mental and physical health conditions.^4^ A recent systematic review found that complex interventions were successful in improving psychological distress for people with MLTCs; however, results for those living with two or more physical conditions were inconclusive. This highlights a need to better understand how to engage this group in interventions.^14^

People with MLTCs commonly experience difficulties with mobility, manage chronic pain and take multiple daily medications, of which side effects become an additional challenge.^15 16^ In addition, those living with a co-occurring mental health condition such as depression, psychosis or anxiety will manage debilitating mental health symptoms, alongside experiences of stigma and shame towards their condition.^17^ This group is found to have a poorer quality of life,^18^ experience loss of social connections and report experiencing poorer healthcare.^3^

Improving knowledge on the treatment, burdens and determinants of people with MLTCs has been set as a priority for global health.^4^ While research on MLTCs is increasing, many studies focus on the prevalence and clusters of conditions,^19 20^ and those exploring the impacts and experiences of having MLTCs lack focus on the co-occurrence of mental and physical health conditions.^21^ Exploring this group’s experiences of receiving treatment is still significantly under-researched.^3^

The ERA study (**E**ffectiveness of group arts therapies compared with group counselling in diagnostically heterogeneous patients: **Ra**ndomised controlled trial) is a randomised controlled trial (RCT) comparing the effectiveness of group arts therapies and group counselling for diagnostically heterogeneous patients in community mental health services (ISRCTN:88805048).^22^ During the recruitment to and running of the trial, we were aware of many trial participants reporting challenges accessing groups due to a range of MLTCs. As trial participants held a range of mental health diagnoses, and all were accessing different types of group therapy, this provided an opportunity to map their experiences within the trial. This study within a trial (SWAT) aimed to explore how living with MLTCs with a comorbid mental health condition impacted the decision-making process, attendance and engagement in community group interventions within the ERA trial.

## Methods

### Study design

We conducted a qualitative SWAT, using reflexive codebook analysis^23^ of semi-structured interviews with a subset of ERA trial participants. Semi-structured interviews allowed us to follow a common structure across interviews, while adapting questions to expand on individual participants’ experiences.

Our epistemological stance was one of contextualism, which acknowledges the importance and role of a person’s individual contexts in shaping their experience.^24^ Within this study, the contexts within which participants’ experiences took place were key to understanding interactions between the impacts of MLTCs, mental health and the specific group environment. We took an experiential approach to the data, with the focus of gathering and further exploring participants’ individual perspectives, while paying attention to their unique contexts.^24^

### Study population and setting

#### Host trial

The ERA trial is a National Institute for Health Research funded, pragmatic two-arm RCT testing the effectiveness of group arts therapies compared with group counselling in diagnostically heterogeneous patients in community mental healthcare in England.^22^ Participants were recruited from NHS community mental health services and all held a primary diagnosis of an International Classification of Diseases, 10th Revision psychiatric disorder^25^ of either an F2 (schizophrenia, schizotypal and delusional disorders), F3 (mood disorders) or F4 (neurotic, stress-related and somatoform disorders). They were also required to score a minimum level of moderate symptom severity (>1.65) on the Brief Symptom Inventory Global Severity Index^26^ to be eligible to participate in the study.

On recruitment to the trial, participants were provided with a participant information sheet which explained the process of participating, the opportunity to participate in an optional semi-structured interview about their experiences as part of the study process evaluation and the option to consent to be contacted about future research related to the trial. Participants were asked to select their preferred group arts therapy at enrolment out of art, music and dance-movement and were randomised to either the arts therapy of their choice or to group counselling. All interventions were delivered as 90-minute sessions, two sessions per week, across 20 weeks.

#### SWAT inclusion criteria

At the point of this SWAT commencing, researchers reviewed the participant database to identify participants who:

1. Were randomised with allocated therapy sessions finished.
2. Provided optional consent to be interviewed.
3. Provided optional consent to be contacted about future research related to the trial.
4. Had a self-reported physical health condition.
5. Had capacity and were willing to participate in an interview.

Potentially eligible participants were contacted by the first author via telephone to explain the purpose of the SWAT and ascertain interest in participating. Those who were interested were given the opportunity to ask questions and time to decide whether or not to take part. Those who wished to participate then indicated a preferred time, date and location for an interview with one of the ERA researchers. Interviews were held according to participant preference, either in person in a private room at a clinical site, via telephone or via Microsoft Teams.

At all contacts, researchers explained that they were researchers who formed part of the ERA study team. Many of the participants had already met these researchers when providing informed consent, completing process measures during the intervention or participating in trial follow-up assessments. Where possible, we assigned researchers known to the participant for the interview to aid rapport.

### Sampling and recruitment

Purposive sampling was used as it is based on the selection of participants through specified criteria.^27^ Participants who were known to have experienced physical health challenges during recruitment and/or their group therapy were collated. Due to practical considerations, two of the trial’s sites were used to recruit participants for the SWAT: East London and Bedford.

Determining the appropriate number of interviews depends on the specific aims, context and scope of each research project.^28^ In this study, to guide our decisions around sample adequacy, we drew on the concept of information power^29^ and used maximum variation sampling in order to capture potential impacts of MLTCs across a broad range of experiences (cross-case analysis^30^). We decided on 20 interviews for pragmatic reasons: this was sufficient to gain a range of group types, diagnoses, gender and attendance characteristics, but not so many to miss an in-depth consideration of the experiences reported within the timeframe of the study.

Participants were approached by the first author (LH) and provided with information about the SWAT’s aims, purpose and what taking part would entail. No participants approached to take part in the study declined.

### Patient and public involvement

The topic guide was developed in discussion with the ERA trial’s Lived Experience Advisory Panel (LEAP). The LEAP is a board of individuals who are experts by experience as mental health service users and carers, and advise on all aspects of the trial design and conduct.

An NHS patient and public involvement lead staff member with MLTCs responded to a call out to review the topic guide and met the first author (LH) to review it. The proposed questions were discussed in detail before making final adjustments based on their feedback. For example, they felt the use of the word ‘multi-morbidity’ was too clinical and suggested specific questions be included on the group locations, rooms and facilities. A further member (JF) assisted with data analysis and interpretation.

### Data collection

20 interviews were conducted by three of the study authors, LH, EMe and EMi, who were all trained and experienced in conducting qualitative interviews in mental health and other vulnerable populations. Interviews were carried out in person, via video call or phone call based on participants’ preferences. Interviews took place across May–October 2023, were audio recorded and lasted between 20 and 70 min (mean=47 min).

The topic guide (online supplemental file 1) was used to explore participants’ experiences of attending (or not attending) their allocated group therapy. Questions explored areas of their physical health conditions, decision-making process of taking part in the ERA trial, experience of the group therapy, challenges they faced and how they were supported with regard to their multiple conditions. Participants were also asked for recommendations to improve interventions in the future for people with MLTCs.

Interviews were expected to last 1 hour and a £20 supermarket voucher was given for participating. Researchers made notes on any standout points and observations after each interview, to inform the analysis and to begin early familiarisation with the data.

To aid description of our sample, we drew on sociodemographic data of age, gender and ethnicity and clinical data of primary mental health diagnosis, group attendance and medication prescribed at postintervention, already collected as part of the trial. Data were input from case report forms into a Microsoft Excel spreadsheet and summarised as counts and percentages. Data from the ERA trial cohort as a whole were included as percentages to aid comparison with the overall trial sample.

### Data analysis

Interviews were transcribed using an NHS-approved transcription service. Transcripts were checked for accuracy, anonymised and imported into NVivo V.12 software for coding.

For pragmatic reasons, to complete the study within the timeframes available in the trial and enable geographical coverage, we involved a team of researchers in the collection and analysis of the data. The team held a range of qualitative experiences: all had experience in conducting research and data collection with mental health populations. This study was the first author’s (LH) first experience of leading and writing up qualitative data and she was supported closely by a postdoctoral researcher with a range of qualitative experience (EMi). One researcher had previous experience of qualitative data collection and coding (EMe) and for one, it was her first experience of coding (EF). Our lived experience author (JF) held extensive experience in qualitative analytic involvement. The last author (CC) held experience in leading a number of qualitative studies embedded within interventional research. To complement this, the study team sought additional review during paper revisions from an experienced qualitative researcher.

We felt that it would be important to ensure a shared team understanding of the data, so we employed analytic methods that typify what Braun and Clarke term ‘reflexive codebook’ analysis,^25^ that is, including steps to ensure consistency of understanding of the coding between members and coverage of themes.

The team began with familiarisation with the data, whereby the first author (LH) read through all transcripts prior to coding. Three researchers (LH, EF and EMi) used NVivo V.12 software to independently apply open codes to an initial selection of three transcripts each. At this point, researchers met to compare and discuss initial categories and codes that were observed so far to gain a sense of the stories unfolding in each interview.

Physical health conditions and symptoms participants reported during their interviews were summarised in order to conceptualise the difficulties they were managing while attending their therapy group, alongside medication data taken from participants’ postintervention follow-up assessment. General topics raised in the interviews, which were outside of participants’ experience relating to both their physical and mental health conditions, were not analysed.

Once most transcripts had been coded, the study team met with the Lived Experience Researcher and LEAP member (JF), who had read two transcripts ahead of the meeting. The team reviewed the independent coding so far and discussed the formulation of the main codes. The team was in agreement on primary topics of interest and JF’s impressions of the data aligned with the researchers’ coding. The team began to refine main themes and subthemes before the first author (LH) continued to code further transcripts and finalise the coding frame.

This SWAT was conducted while the ERA trial was underway; therefore, participants took part in this study at varying points of their post-group trial participation. In many instances, participants had previously been recruited into the study or completed lengthy follow-up assessments with the interviewers, which may have aided rapport, but also had the potential to influence what participants felt able to share. The research team was already aware that participants’ MLTCs were causing significant challenges in their group engagement and attendance; therefore, assumptions may have been made about the issues that would be raised in the interviews. These assumptions were discussed and reflected on during analytic meetings. Team members had varying personal experiences of mental illness or long-term physical health conditions alongside varying professional backgrounds from psychology, counselling and arts therapies.

The team decided to map out key uncertainties participants were managing across their participation from a first-person perspective to help reconfigure the data, which were mapped out onto a timeline. Codes were revisited to further refine themes recursively and were discussed at length with the whole research team until final themes and subthemes were agreed on. Three interviews were then coded by two researchers (CC and EF) into the final coding frame to check its validity and coverage.

## Results

### Participant demographics and clinical information

Table 1 reports^1^ demographic information, mental health conditions, group therapy allocations and attendance levels of the 20 SWAT participants alongside a comparison with wider trial participants. Table 2 reports the medication prescribed to participants at the postintervention follow-ups. All participants were on medication for their mental health. Antidepressants were the most commonly prescribed medication, followed by sedatives and drugs to lower cholesterol. Participants ranged from taking 1 to 16 different medications (mean=7).

**Table 1:** SWAT participant and overall trial demographic information, mental health conditions, therapy groups attended and level of attendance

Gender	SWAT sample (n=20)		ERA trial sample (n=425)
	N	%	%
Male	10	50	45
Female	10	50	55
Ethnicity			
White British	7	35	50
Black/Black British Caribbean	3	15	17
Asian/Asian British Indian	2	10	19
White other	2	10	4
Other mixed	4	20	8
Prefer not to say	2	10	2
Primary diagnosis			
F2 Schizophrenia	2	10	29
F3 Depression	8	40	42
F4 Anxiety	10	50	29
Age range			
40–49	2	10	Mean age=44 (SD 13.11)
50–59	9	45	
60–69	8	40	
70–79	1	5	
Group therapy attended			
Art therapy	7	35	36
Music therapy	5	25	10
Group counselling	5	25	47
Dance movement therapy	3	15	7
Level of attendance			
Non-attendance (no sessions attended)	2	10	29
Low group attendance (less than 50% of sessions attended)	6	30	34
Medium group attendance (50%–75% of sessions attended)	6	30	19
High group attendance (over 75% of sessions attended)	6	30	18

ERA, *E*ffectiveness of group arts therapies compared with group counselling in diagnostically heterogeneous patients: *Ra*ndomised controlled trial; SWAT, study within a trial.

**Table 2:** Medication prescriptions at postintervention timepoint for participants

Primary medication use (number of unique participants)	Medication class	Participants (n)
Antipsychotics (9)	Typical	1
	Atypical	8
Antidepressants (16)	SSRI	9
	SNRI	2
	NaSSA	4
	Tricyclic	3
Anticonvulsant (7)		7
Sedatives (10)	Antihistamine	8
	Benzodiazepines	3
	Non-benzodiazepine hypnotics	3
Analgesics (6)	Opioid	6
	Other analgesics	1
Anti-inflammatory (1)	NSAID	1
Anticoagulant (1)		1
Antispasmodic (5)		5
Movement disorders (1)	Dopamine agonist	1
Gastric (9)	Proton pump inhibitor	9
	H2 receptor agonists	1
	Stimulant laxatives	1
Cholesterol (9)	Statins	8
	Selective cholesterol-absorption inhibitors	1
Hormones/HRT (3)		3
Hypertension (7)	Alpha blockers	4
	Beta blockers	3
	Calcium channel blockers	2
	ACE inhibitors	2
	Xanthine oxidase inhibitors	1
Immune system/Asthma (5)	Corticosteroids	5
	LTRAs	1
	Short acting beta-2 adrenergic agonist	2
Diabetes (4)	Sulfonylureas	2
	SGLT2 inhibitors	3
	DPP4 inhibitors	2
	Biguanide	2
Diuretic (2)		2
Complementary (1)	Homeopathic remedies	1
	Mistletoe injection (cancer)	1
Nutritional deficiency (3)	Vitamins	3
	Iron	1
	Magnesium	1
	Oral nutritional supplements	1

DPP-4, dipeptidyl peptidase-4; HRT, hormone replacement therapy; LTRAs, leukotriene receptor antagonists; NaSSA, noradrenergic and specific serotonergic antidepressant; NSAIDs, non-steroidal anti-inflammatory drugs; SGLT2, sodium–glucose cotransporter-2; SNRI, serotonin–norepinephrine reuptake inhibitor; SSRI, selective serotonin reuptake inhibitor.

### Physical health conditions and symptoms reported

#### Physical health conditions

**Figure 1:**
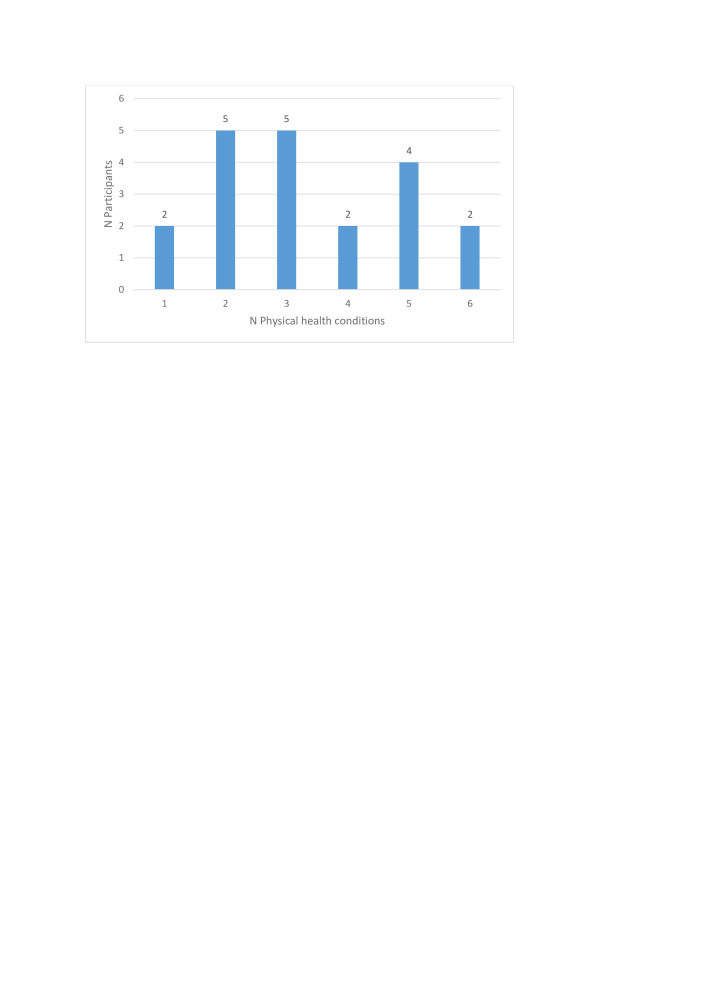
Number of physical health conditions per participant.

In total, 31 different physical health conditions were reported by participants. The number of physical conditions per participant ranged between one and six, with a mean of 3, as detailed in figure 1.

Table 3 lists all physical health conditions reported by participants and the number of participants who have each of them. Spinal disc damage (9) and arthritis (9) were most commonly reported and having limited mobility, severe pain and using a walking aid or wheelchair were frequently described as symptoms by participants with these conditions.

**Table 3:** List of physical health conditions reported

Physical health conditions reported	n
Spinal disc damage (herniated discs, slipped discs, spondylosis, lumbar degenerative disease)	9
Arthritis (rheumatoid arthritis, osteoarthritis)	9
Diabetes	5
Fibromyalgia	4
Digestive disorders (including IBS)	4
Stroke	2
COPD	2
Asthma	2
Cancer (in-treatment)	2
Cancer (in remission)	2
Heart condition	2
Incontinence	2
Gynaecological condition	2
Migraines	2
Long-COVID	2
Fracture	1
Chronic pain	1
Tendonitis	1
Osteoporosis	1
Muscle atrophy	1
Angina attacks	1
Lipoedema	1
Degenerative bone disease	1
Chronic fatigue syndrome	1
Carpal tunnel syndrome	1
Insomnia	1
Haglund’s deformity	1
Tendonitis	1
Visual impairment	1
Blood clots	1
Gout	1

COPD, chronic obstructive pulmonary disease; IBS, irritable bowel syndrome.

Diabetes (5), fibromyalgia (4) and digestive conditions (4) were also reported, with symptoms of pain and fatigue often reported with them. Hypertension was not reported despite there being evidence of this in the sample from the medication prescribed.

#### Physical health symptoms

Participants described a total of 34 symptoms of their physical health conditions, which were impacting their day-to-day lives. Difficulties with managing pain and fatigue led to participants infrequently leaving their home, spending a lot of time in bed and feeling isolated. Most participants took multiple daily medications for both their mental and physical health conditions, which they described as exacerbating feelings of tiredness, feeling weak and having trouble remembering things.

Some participants had daily support from carers or family members, who assisted them with tasks such as washing, dressing and cooking. Participants preferred to avoid using public transport due to difficulties with walking far and needed regular opportunities to sit down to manage their pain and fatigue when they did leave their homes. Becoming disoriented, confused and dizzy was also described as symptoms that impacted participants when travelling, further adding to their inclination to stay home.

## Findings

### Timeline of challenges

**Figure 2:**
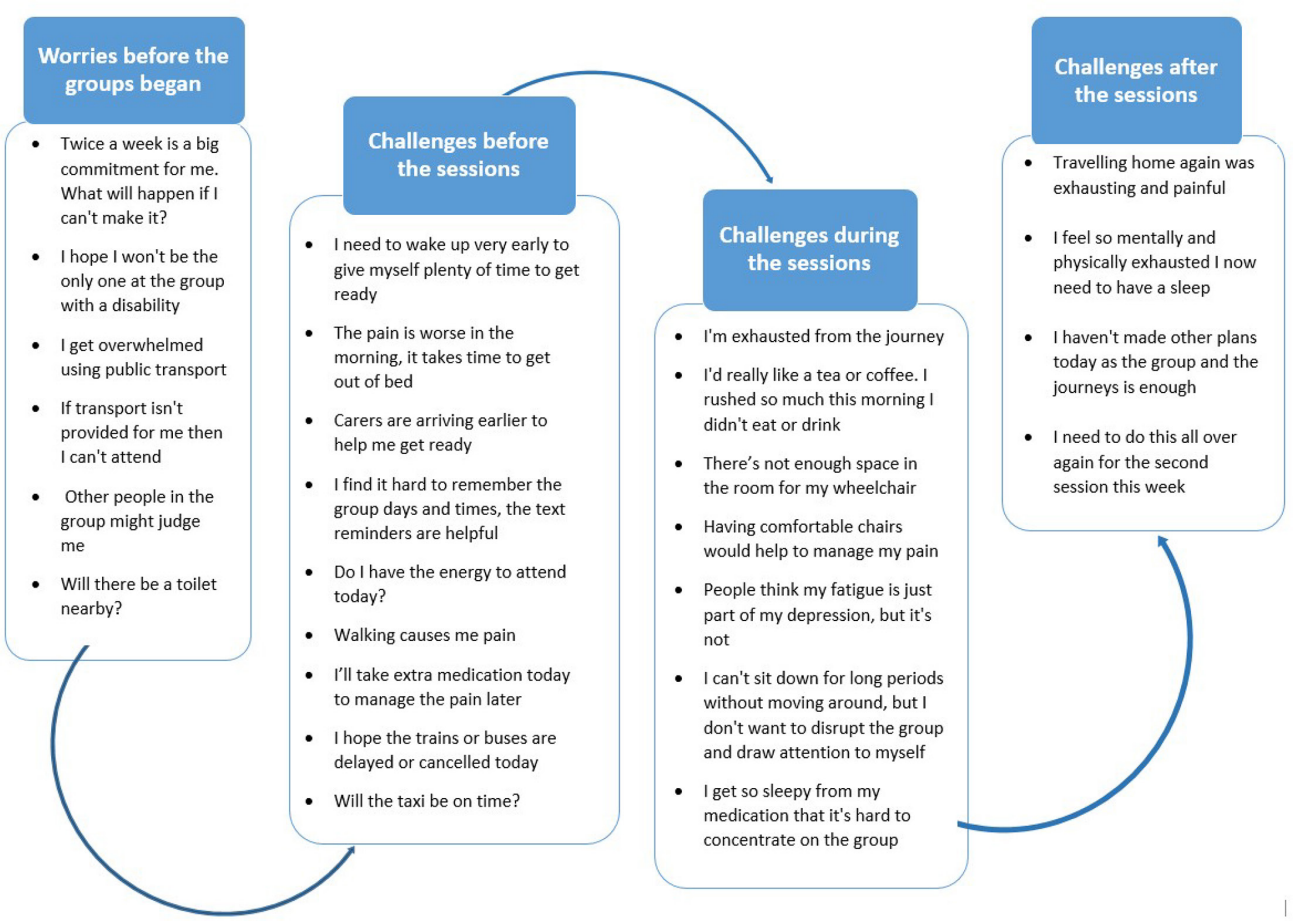
Timeline of worries and challenges expressed by participants.

Participants described many concerns that arose when deciding to take part in the ERA trial, as well as worries related to their MLTCs when attending sessions week to week. On reviewing the themes, the study team found it useful to map these worries and challenges onto a timeline in order to see how concerns were impacting participants over time, as outlined in figure 2.

### Themes

Six main themes were constructed from the interview data analysis. The themes, their subthemes and a summary are outlined in table 4.

**Table 4:** Overview of themes and subthemes

Theme	Theme overview	Subthemes
**1. MLTCs influenced arts modality choices and goals** “It will always be what am I capable of doing”	The arts modality choices participants made were often based on what participants felt physically capable of and what they had previous experience of. Attending the dance-movement group was seen as an opportunity for a physical health as well as a mental health intervention	1.1 Feeling capable; arts modality choices 1.2 Hopes of the dance-movement group being a tool to improve physical as well as mental health
**2. Importance of planning ahead in order to be organised** “Everything I do is scheduled”	It was important for participants to have structure and routine by planning ahead and pacing themselves. This organisation enabled them to account for their additional physical health needs in advance and ensure necessary support was in place before committing to attending	2.1 Routine and pacing self 2.2 Planning carer support and caring responsibilities 2.3 1–1 therapist meeting enabled people to feel informed ahead of the group 2.4 Venue and location considerations
**3. The journey loomed over participants** “The big unknown was what would happen during the travel period”	Travel to the groups was dreaded. The journey was an unknown element of their participation which participants could not control. Travelling put participants under physical and mental strain, which also had an impact on their ability to participate in and their experience of group therapy	3.1 Travel to the group was a factor they could not control 3.2 Groups were far from home 3.3 Offer of taxis was vital to attending 3.4 Impacts of travelling on symptoms 3.5 Impacts of the journey on the group therapy
**4. The impact of MLTCs on group attendance and participation** “I didn’t want to spoil the group by sitting down”	Physical and mental health symptoms were interlinked and exacerbated one another. During group sessions, participants used a variety of ways to manage physical health symptoms; some were conscious of being judged by others for doing so. Implications of attending twice a week and attending morning sessions made attending challenging and often impacted their ability to attend. Participants felt disappointed and ashamed when their MLTC prevented them from attending. Participants required flexibility and understanding from the group therapists in order to adapt their participation depending on their presenting difficulties	4.1 Problems being doubled 4.2 Managing physical symptoms during the group 4.3 Adapting participation to make adjustments for physical symptoms 4.4 Negative view of self and disability 4.5 Two sessions a week were hard to maintain 4.6 Morning sessions were hard 4.7 Feelings of disappointment and shame for non-attendance
**5. The group was important and valued** “The group was a lifeline”	Groups offered a number of opportunities to holistically address mental and physical challenges and participants were motivated to attend. The group therapy provided a reason to regularly leave their house and an opportunity to connect with other people who were like-minded. Participants particularly valued meeting others with similar MLTCs and challenges. For most, the group became an important fixture in their week which they enjoyed and was held in high regard	5.1 Enjoyment and fun 5.2 A reason to leave the house 5.3 Other group members having MLTCs was liberating 5.4 Support from facilitators and group members
**6. Determination and fighting to get what I need** “I wouldn’t allow my health to get in the way”	Despite the challenges detailed, participants were determined to attend the group and were committed to seeing it through. However, many described having to fight for their MLTC to be taken into account both in their wider care and in the group. Therapists did not always fully understand the impact of the MLTC on participants, with some failing to provide the appropriate support	6.1 Committing to attend despite challenges 6.2 Needing to fight to receive treatment 6.3 Lack of understanding/support from the therapist

MLTCs, multiple long-term conditions.

### Theme 1: MLTCs influenced arts modality choices and goals

#### Subtheme 1.1: Feeling capable; arts modality choices

The choice of arts modalities participants made between art, music and dance-movement was largely based on what they felt physically capable of doing. Being able to sit down during the group was mentioned as an important consideration for many participants’ arts modality choices, as was their previous experience of the modality.

Because I knew with art, you sit down basically. You sit down and do your projects. It will always be what am I capable of doing. And out of those three I picked the art because I knew it’s laid back and it’s the sort of people I like. (2 conditions, medium attender, art therapy)

#### Subtheme 1.2: Hopes of the dance-movement group being a tool to improve physical as well as mental health

For some participants, arts modality choices were perceived as an opportunity to improve physical health symptoms, with a hope that the dance-movement group could be a physical health intervention as well as a mental health intervention. “Well I particularly wanted with, erm, dance therapy because it helped me like, er, physical strength as well a little bit, together with the mental things as well. So that’s why I chose that one” (2 conditions, low attender, dance-movement therapy).

### Theme 2: Importance of planning ahead in order to be organised

#### Subtheme 2.1: Routine and pacing

It was important for participants to plan ahead for their participation to ensure they were able to attend. Some explained that maintaining a routine was vital as they had to limit their commitments in order to manage their pain and fatigue.

Previously to this I was really fit but now I have to manage my day in terms of, you know, being very lethargic. I can’t manage more than a few things a day, so everything I do is kind of scheduled. (5 conditions, low attender, art therapy)

#### Subtheme 2.2: Planning carer support and caring responsibilities

Participants appreciated having information in advance, which allowed them to organise adjustments required in their routine and their care. For one participant, they needed to plan for their own carers’ responsibilities.

For some, this involved planning for carers or family members to be home before the group to support them with getting ready, travelling and altering medication timings. “And my carers reminded me, ‘You’ve got your group today’ You know, and I say, “Oh yes, yes, yes I have my group’. (…) they’d come early to get me ready so I could attend my group” (6 conditions, high attender, group counselling).

#### Subtheme 2.3: 1–1 therapist meeting enabled people to feel informed ahead of the group

Participants had a 1–1 meeting with the group therapist prior to the group starting, which was vital for some to attend, as they could clarify the group locations and timings so they could be prepared. This was also an important opportunity to discuss perceived problems and seek reassurance on what to expect from the group.

I think that’s critical, without the one to one I wouldn’t have engaged (…) they managed to allay my fears really. (5 conditions, medium attender, art therapy)

It was highlighted that this initial meeting was a missed opportunity to have been asked directly about their physical health needs. “Before we start our therapy people with health conditions need to be, you know, asked is there anything that we can do for you?” (4 conditions, medium attender, group counselling).

#### Subtheme 2.4: Location and venue considerations

The group venue and facilities were important factors to consider when deciding to participate and the venue being close to a bus stop was helpful. For participants with incontinence and digestive disorders, accessibility of toilets was important.

They are things that put me at ease, put it that way. If I know everything’s there, I don’t worry. If I go to a place and I don’t know where the toilets are, just in case, I start putting problems there in front of me before they actually exist. (2 conditions, medium attender, art therapy)

### Theme 3: The journey loomed over participants

#### Subtheme 3.1: Travel to the group was a factor they could not control

Travel to and from the groups was a significant concern for participants before their group began. “I think the key thing was just would I be able to get there and, you know, to the venue, how tiring it would be” (2 conditions, medium attender, music therapy).

Travel to the groups was dreaded due to the unpredictability of external factors such as accessibility, train delays and arriving late to the session.

When all these transportation strikes started to happened, then I was left stranded and then I had to walk all the way (…) and that was another half an hour walk on top of the journey, which I shouldn’t have been walking to at all because of the back and leg problem. (5 conditions, high attender, music therapy)

#### Subtheme 3.2: Groups were far from home

Due to the impacts of the journey, participants would have preferred their group therapies to be closer to home. “My gosh, having somewhere closer. The journey itself tired me out. And then the sessions were always good but it was the journey” (6 conditions, high attender, group counselling).

#### Subtheme 3.3: The offer of taxis was vital to attending

Many participants were unable to travel independently because of their physical health symptoms. This resulted in participants being provided with a taxi when attendance would otherwise not have been possible, for which they expressed great appreciation. “They made it accessible and it was a God send really because without it I wouldn’t have been able to commit and obviously go through that journey that I did through the group and through the study” (5 conditions, medium attender, art therapy).

#### Subtheme 3.4: Impacts of travelling on symptoms

Participants worked hard to attend the groups. Travelling placed them under physical and mental strain.

So a slow walk, you know because obviously because of my back and everything. But the other thing (…) I get disorientated. (…) So sometimes I just feel you know like I don’t know my way. And then I start to panic and I start to get anxious and stuff like that. (4 conditions, medium attender, group counselling)

#### Subtheme 3.5: Impacts of the journey on the group therapy

The therapy groups were also impacted by the strain of travelling, due to the need to spend time decompressing and recovering from the journey once participants arrived. In addition, lateness to the groups due to difficult journeys, including late taxis, created further interference.

You never had enough time to talk about what you wanted, because too much time was being spent on the frustration of getting there. Also the trepidations of getting home again after it. (5 conditions, high attender, music therapy)

### Theme 4: The impact of MLTCs on group attendance and participation

#### Subtheme 4.1: Problems being doubled

Participants detailed how having physical as well as mental health conditions results in problems being doubled. *“*If you’ve got physical problems then navigating the journey is going to be a physical problem. If you’ve got mental health and physical problems, then you’ve got two problems” (5 conditions, high attender, music therapy).

Their MLTCs were often described as being interlinked and symptoms would exacerbate each other in a cyclical manner. “It’s clearly a cycle. I mean the more I- I get extremely stressed about going to places because I’m worried about my IBS. And the IBS gets worse when I’m stressed” (5 conditions, low attender, art therapy).

#### Subtheme 4.2: Managing physical symptoms during the group

During the groups, participants needed to find ways to manage their pain. Getting up to walk around or stretch, leaving the room temporarily or moving to get more comfortable in their seat was commonly described.

The chairs would be set around for you, but I got ones that had arms on because I have the pains in my legs, my bum and everything. If you use the arm of the chair then you can kind of squat to the side. (3 conditions, high attender, group counselling)

For some, they felt guilty for interrupting the rest of the group when they needed to.

You feel guilty if you’re going to interrupt like other people. So I would feel guilty, which one day I did walk away for a while, but they asked me, “You all right?” (…) I just want to be like a normal person, I don’t want to have people look at me in certain way. (1 condition, low attender, group counselling)

Medication side effects also impacted participation as well as engagement with other group members.

I’m trying to fight to stay alert and the medication would often creep in and make me feel tired. (…) So where I like to give people my full concentration sometimes I didn’t feel that I could concentrate any more. (6 conditions, high attender, group counselling)

#### Subtheme 4.3: Adapting participation to make adjustments for physical symptoms

Some participants required adjustments during their group to support their participation. Dance movement participants could engage while sitting to manage their pain.

*“*Well when I was up moving around and then I got pain I just went and sat down. And sat down for a while and then I got back up again. And, you know, took part. And yes, it was very good” (2 conditions, medium attender, dance-movement therapy). This participant felt at ease when they found other group members sat down for periods too. “I thought, oh well I’m not the only person. I thought at first I didn’t want to spoil the group by sitting down, you know? But that was more in my own mind than anything else” (2 conditions, medium attender, dance-movement therapy).

In the art therapy groups, participants appreciated options such as taking artwork home to continue and bring back, easing pressure during the session, or continuing work from previous sessions instead of starting something new when feeling overwhelmed. A music therapy participant explained that they would select instruments which were light and could be held with one hand or which could be held on their lap, highlighting how physical ability influenced their engagement.

#### Subtheme 4.4: Negative view of self and disability

Participants were self-conscious of their physical conditions, feeling inferior to others and concerned that they would be judged by other group members.

Because I feel that people look at me (…) and they think, you know, I’ve got a brain disorder or something. I just feel judged by people and that people, so going into a group is quite difficult for me. (6 conditions, high attender, group counselling)

#### Subtheme 4.5: Two sessions a week were hard to maintain

Attending the group twice per week was challenging and a factor they considered when deciding to participate at all, knowing this would be a strain. Many participants stated that they seldom leave their homes, so this two times per week attendance was a significant commitment for them. Participants tended to favour one longer session per week. “The back issues, the mobility issues, the bipolar, in particular, and the insomnia, and the stomach, dietary (…) Coming in twice and getting up very early to do it was a strain” (4 conditions, high attender, art therapy).

#### Subtheme 4.6: Morning sessions were hard

Groups scheduled in the morning were a difficulty for most participants. They explained how pain is worse in the mornings and they require time for pain to ease, to take medications and to get themselves ready. Sessions held in the afternoon were much preferred.

I get really worse in the morning. I find it very difficult to get out of bed because my back pain is really severe. I have to hold myself and then swing around, and then the pain is very intense. So that takes a bit of time for me to settle down. (5 conditions, medium attender, art therapy)

#### Subtheme 4.7: Feelings of disappointment and shame for non-attendance

Some participants described occasions when they had to miss group sessions due to illnesses, increased pain or having other health appointments to attend. They felt disappointed and guilty for letting others down when this occurred and missed being at the group.

Two participants who did not manage to attend any group sessions explained that this was due to their physical health at the time, and both expressed their disappointment at not being able to attend. For one participant, they explained they were experiencing “one illness after another” (3 conditions, non-attender, art therapy), which also exacerbated their mental health symptoms. They expressed appreciation for the contact they received from the group therapists who checked in with them regularly through the duration of the group, but the guilt they felt for not attending added an additional strain: “But that guilt creeps in because I don’t like to let people down. So, then you’ve got to deal with that as well” (3 conditions, non-attender, art therapy).

### Theme 5: The group was important and valued

#### Subtheme 5.1: Enjoyment and fun

The groups offered an opportunity for enjoyment and fun with others, expressed particularly among participants who attended arts modality groups.

The enjoyment of the group gave some a distraction from their pain *“*When you enjoy doing something, the pain is there but it’s not that severe because your mind is so occupied*”* (3 conditions, high attender, music therapy).

#### Subtheme 5.2: A reason to leave the house

Many participants mentioned being grateful that the group provided an opportunity to regularly leave their homes. One participant, who is a wheelchair user and relies on a taxi to attend, did not discover that a group session had been cancelled until they had arrived at the building. They explained that, nevertheless, this was still an opportunity for them to go out.

I came for the group sessions and then they said, “There is not, it’s been cancelled.” Then I said, “Oh, I didn’t get any text or anything. (…) But it was all right for me because it was an excuse for me to go out, so. (1 condition, low attender, group counselling)

#### Subtheme 5.3: Other group members having MLTCs was liberating

Having other people in the groups who were experiencing both physical and mental health problems was ‘liberating’ and put participants at ease.

That was quite liberating. Because I wasn’t the only person who had a physical disability and I wasn’t the only person that had, you know, used a wheelchair or needed to use crutches. And I wasn’t the only person who had carers. That meant a lot to me. (6 conditions, high attender, group counselling)

A participant explained that she could not identify with anybody else in her group after the only other group member with MLTCs left. “There was only one other woman who had multiple condition (…) But she didn’t stay and that’s one person that I could seriously identify with” (2 conditions, low attender, art therapy). This participant felt able to relate to them in a way they could not with other group members: “I just could relate to her. It was like she could open up, then I could feel strong with her and I felt that I wasn’t the only one in the group” (2 conditions, low attender, art therapy).

In addition to feeling understood and at ease with the group member with MLTCs, this participant’s physical health condition was misunderstood by those without: “Then coming back to the group, it was challenging afterwards because I couldn’t explain to them about my fatigue, and they kept seeing it as, oh, it’s your depression” (2 conditions, low attender, art therapy).

#### Subtheme 5.4: Support from group therapists

Most participants gave positive accounts of the support they received from their group therapists and group members. Therapists were considerate of participants’ needs once they were made aware of ways in which they could provide help, such as meeting participants from their taxi to support them walking inside and helping to adjust sitting positions to aid comfort.

One participant would begin the journey to the group but then needed to go home again due to a sudden irritable bowel syndrome flare-up, which group therapists were supportive about.

It was also encouraging to be told ‘we would like you to come but if you can’t that’s okay’ because I feel a sense of failure very often. And sometimes I’d get there very late and to be told ‘Good we’re glad that you managed to get here at all. (5 conditions, low attender, art therapy)

### Theme 6: Determination and fighting to get what I need

#### Subtheme 6.1: Committing to attend despite challenges

Despite the numerous challenges participants encountered in attending and engaging in their group, they were nevertheless determined to persevere and attend. Working so hard to attend the groups reinforced their commitment and fortitude to see it through.

I wouldn’t allow my health to get in the way of me receiving something which the therapy could. So once there I tried hard to get as much out of it as I could. And just try to take my mind off the pain and off my situation. (6 conditions, high attender, group counselling)

#### Subtheme 6.2: Needing to fight to receive treatment

This determination was also applied more widely to their general healthcare, in which many participants explained that they needed to ‘fight’ to be seen and heard in services, as they often felt overlooked, which left them feeling drained.

It’s debilitating mentally the fact that I know that I’ve got to fight and I want to, you know- And it’s only people like me that recover from physical, you know, or can get better or can improve on their physical disabilities because I’m determined. (5 conditions, medium attender, art therapy)

Furthermore, some participants disclosed how their physical health conditions had been misunderstood and overlooked as mental health symptoms by clinicians, leading to delays in receiving diagnoses, illuminating the complex interplay between their physical and mental health.

#### Subtheme 6.3: Lack of understanding/support from the therapist

At times, therapists lacked understanding of how having MLTCs was impacting participants’ attendance and engagement. Providing some direction on selecting instruments in music therapy, which would put participants under less physical strain, would have been helpful. One participant felt pressured by the therapist to return to the group, as they continued to contact her after she had already declined.

A participant who declined the offer of a taxi at the beginning of their group expressed disappointment that the therapist did not re-offer this support option sooner: “Clearly I’m struggling, perhaps they should just say to me, look, you need to take a taxi, because I’m quite stubborn and I'll kind of put up and persist with a problem rather than asking for help” (5 conditions, high attender, music therapy).

## Discussion

Results from this study within the ERA trial uncovered numerous challenges participants with MLTCs encountered when attending a therapy group in the community. Six themes outlined the impacts of managing co-occurring physical and mental health conditions when attending the group. Although the physical health conditions experienced by participants were wide-ranging, the difficulties faced with attending their group were very similar and their mental and physical symptoms were often interlinked.

The challenges and worries participants encountered across their participation were broad and they overcame many challenges in order to attend. Participants worked hard to uphold their commitment to attend their therapy group. The decision to take part tended to be based on feeling capable and prepared. Consistent with previous research with participants with mental health conditions, it was important to feel sufficiently informed and have opportunities for autonomy when attending psychosocial interventions^12^ such as making adaptations to their participation. Participants had to work hard to attend their group and the challenges they encountered demonstrated how having co-morbid physical conditions further compounded this necessity to feel fully informed in order to prepare and alter routines for new commitments. It is recommended that therapists pay attention to physical issues and needs, to ensure appropriate support can be provided to improve attendance and their group experience. Organising a 1–1 in-person meeting prior to groups starting provides a fundamental opportunity to ask group members about their physical health needs and ways in which they could be supported to attend and engage during groups.

Participants’ arts therapy choices being largely based on what they felt capable of aligns with previous research by Millard *et al*,^31^ who also found that participants’ choice of arts engagement felt limited due to their physical ability. Although some used their arts choice to challenge themselves physically, most stayed close to a choice that felt safe and manageable. For arts therapies services, making known the adaptations that can be made to modalities that are perceived as inaccessible by people with MLTCs could broaden their felt opportunities and normalise doing so. Furthermore, participants valued when other group members had MLTCs too, as they felt more at ease and understood. Therefore, therapy groups offered exclusively for people with MLTCs could improve confidence when managing symptoms and reduce the fear of judgement and negative view of themselves, as found in this study.

Physical and mental health symptoms being misunderstood by group members due to being so closely interlinked was also experienced in their wider healthcare treatment. Diagnostic overshadowing is the misattribution of physical health symptoms as symptoms of a pre-existing mental health condition, which can result in delays in physical conditions being diagnosed^32^ as reported by some participants. Diagnostic overshadowing is hypothesised as a possible factor in the health inequalities faced by people with MLTCs^33^ and requires an increased focus in the literature so improvements can be made to the standard of healthcare this increasing population receives.

Participants describing how they ‘fight’ for their healthcare highlights a vital need for more support in navigating the multiple healthcare systems they are bound to rely on. The sharp rise in the occurrence of MLTCs has brought calls to move away from fragmented and singular care delivery and move towards integrated care.^34^ The cyclical way in which mental and physical conditions are interlinked and can exacerbate one another, as found in this SWAT, further supports calls for a joined-up healthcare system, where the whole person is treated, and not individual conditions across multiple services.^18^ Treatment burden refers to the work people have to put in, in order to access healthcare, and how this effort impacts them.^35^ For future community-based interventions, results from this study recommend that consideration be taken over group timings, supporting transport needs and reviewing accessibility of venues to ease treatment burden for this group. Similarly, within RCTs, consideration should be given to the physical needs of participants, including mobility, fatigue and levels of pain in terms of all participant processes and data collection. Having intervention preferences taken into account appeared particularly important for this group. Similarly, the consistency of researcher contact appeared to aid rapport and add to existing evidence for improving retention in RCTs, especially in mental health.^36^ We would recommend collecting comprehensive data on both physical and mental health diagnoses held, and considering in advance adaptations, such as minimising travel and accessibility, alongside potential additional burdens.

### Strengths and limitations

Results from this study must be considered in the context of its limitations. Participants completed interviews at different stages post their group therapy ending; some were within weeks, while some were around a year later. This range in length of time since the group ended may have influenced the experiences being described during interviews, with more time being passed resulting in remembering less accurate details.

Interviews ranged widely in length, with the shortest interviews being the two non-attenders, who had less to discuss in the absence of the group experience to reflect on. The majority of SWAT participants were recruited from East London, and very few were from the Bedford site. The travel issues that arose for participants may not be transferable outside of urban areas. In more rural areas, transport links may be even less accessible and travel to groups could bring additional considerations.

The sampling of participants is a strength of this SWAT, which ensured representation of types of group therapy attended, high, low and non-attenders, and a range of mental health diagnoses. We were only able to sample participants of an older age (43–77), which is representative of the ages of ERA study participants and is expected with physical health difficulties commonly worsening with age^4^; however, we may have missed the experiences of younger people with MLTCs. Similarly, only two participants held a schizophrenia or related disorder diagnosis and thus, we may have missed more nuances relating to experiences with MLTCs and psychosis.

Our participants predominantly held musculoskeletal or mobility-related conditions, which may have driven the first three themes of our findings. Similarly, while loneliness and social isolation are common mental health risk factors, themes 5 and 6 demonstrate how physical conditions can precipitate and maintain this, alongside the benefits of groups in motivating social contact.

Participant physical conditions were based on self-report and not on a structured assessment or medical record. It is therefore possible that we missed capturing less prominent conditions and their impacts. The summary of medication received suggests that a number of participants also had hypertension but did not disclose this, possibly due to the absence of or masking by more troublesome symptoms. However, apart from this, participant-reported problems tended to align with the medication received.

It is important to acknowledge that no participants declined to participate in this study out of those approached. This shows that sharing these group experiences in relation to managing MLTCs was important to participants and they wanted their voices to be heard, especially for the two participants who participated despite having never attended their group therapy.

### Future research

Future research can build on the findings in this SWAT to develop implementable ways to improve accessibility and attendance to community therapy groups for those with MLTCs. Clinicians need to improve their understanding of how to support MLTCs when designing and delivering mental health interventions; therefore, future work could develop co-produced guidelines in collaboration with service users for improved accessibility and engagement in community-based interventions.

The delivery of community-based therapy groups exclusively for people with MLTCs, particularly arts therapies such as dance-movement, would be valuable to investigate the mental and physical health benefits of, which participants in this study explained they hoped would produce dual benefits for them.

### Clinical implications

Based on participants’ recommendations and the wider findings from this SWAT, the following recommendations are outlined in box 1 for clinicians of future community group interventions to consider:

Box 1 **Recommendations for future community groups**

**Recommendations when planning future community interventions**

*Organisation ahead of the group*

- Meet 1–1 with group members prior to the group starting to discuss concerns and enquire about their physical health needs.
- Offer venues with good transport links, close to bus stops.
- Inform participants about the group venue, available facilities and public transport links as far in advance of the group starting as possible.

*Timings of the group*

- Plan for afternoon sessions.
- One session a week is sufficient as a commitment.
- Have a break in the group with refreshments available.

*Support from therapists*

- Send a text reminder ahead of every session.
- Where possible, have multiple group members with MLTCs, enabling people to feel better understood.
- Allow for adaptations to engagement for group members dependent on their presenting symptoms.
- Query group members felt the ability to contribute and engage at the beginning of each session.
- Support people with feelings of guilt, failure or regret when unable to attend.

*Transport*

- Obtain a budget to offer a provision of transport to those who are not able to travel independently.
- Re-offer providing transport across the group as symptoms may change.

*Accessibility considerations*

- Comfortable chairs help with managing pain during the group.
- Room sizes to be big enough to allow wheelchairs to move freely.
- Consider ventilation so rooms have a window that can open.
- Seating outside for if people arrive early.
- Ramps outside the building.
- Toilets are close to the room on the same floor, with a disabled toilet.

MLTCs, multiple long-term conditions.

## Conclusion

This study has outlined the large impact that MLTCs have on the decision-making processes and capacity to engage in community therapy groups. The scale of this impact can go unrecognised by healthcare professionals and researchers alike. While the challenges to attending can be great, participants in this study described how group attendance specifically helped in managing aspects of the MLTC alongside mental health, including motivation to leave the house, opportunities for socialisation and a means of reaching one’s own goals relating to the MLTC. Researchers are recommended to collect data on MLTCs held in mental health studies, alongside accommodations and adjustments that may be necessary for research participation. Clinicians are recommended to proactively enquire about MLTCs and their impact when designing and assessing people for community group interventions and to consider inclusion of multiple people holding MLTCs in their overall group composition.

## Supplementary material

10.1136/bmjopen-2025-103035 online supplemental file 1

## Data Availability

Data are available upon reasonable request.
