# Supplementary material for: Fighting for care: how can we better support people with multiple long-term conditions who are accessing community mental health groups? A qualitative interview study within a UK arts therapies trial
Source: BMJ Open. 2026 Mar 3;16(3):e103035. doi: 10.1136/bmjopen-2025-103035 (PMC12959026; doi:10.1136/bmjopen-2025-103035)
Supplement: online supplemental file 1 [file bmjopen-16-3-s001.docx]

**ERA Multiple Long-term Conditions Interview Topic Guide**

| **Date of interview:** |  |
| --- | --- |
| **Participant ID Number:** |  |
| **Participant group attended:** |  |
| **Participant diagnoses:** |  |
| **Participant attendance:** |  |
| **Researcher initials:** |  |

Is now still a good time for you, this should take around 1 hour, are you in a private space?

Thank you for agreeing to take part in this interview. We are conducting the interviews to understand more about the impact of having multiple conditions on attendance and engagement with the ERA arts therapies and counselling groups. You have been invited to participate because you have a physical condition as well as your mental health condition and we are aware that you experienced some difficulty in attending or engaging in the group due to your physical health. We would be really grateful if you could tell us, in as much detail as possible, how this affected your ability to participate in the groups. It’s up to you how much you share so please only tell us as much as you feel comfortable with. If you need to take a pause or stop the interview at any time, please just let me know.

We are video recording the interview but you can turn off your camera is you prefer, as we will only use the audio recording to transcribe and analyse what you say, to look for themes. One of the aims is to look at supporting people with multiple conditions. It may be linked with your mental health condition or it may not be linked, anything you have to say will be really useful and will provide us with information to create guidelines and improve services in the future. We will publish a paper that summarises the findings from this research, it will not be possible to identify you in any of the published material. We hope that these publications will help to make therapy groups more accessible for people with multiple conditions.

1. Please tell us about any physical health conditions that affect you
   1. Have you been given a diagnosis for this? If so what it is?
   2. Do you have any other difficulties?
   3. How do they impact you on a day to day basis?
2. What was it like for you deciding whether to take part in the ERA study?
   1. Did you have to consider any particular factors related to your physical condition?
   2. Was your physical health a consideration when deciding which arts group (music, dance or art) you would prefer?
3. Did you have any worries about the groups before you went?
4. Can you tell me a bit about the ERA group you attended?
   1. Which arts therapies/counselling group was it?
   2. How did you find attending the group?
   3. What was your experience like?
5. Did you have any problems with attending the groups?
   1. Transport
   2. Having energy to go
   3. Motivation
   4. Cost
   5. Other responsibilities, e.g. caring duties
   6. Other medical appointments
   7. Remembering the session times/days
6. Did you have any problems once you were at the groups?
   1. Pain
   2. Fatigue
   3. Focus
   4. Access to facilities (tea/coffee/toilets/group room/ventilation
   5. Lack of support and understanding (group members/therapists)
7. Was there anything that was in place that helped you to attend?
   1. Flexibility with attending
   2. Taxis/transport
   3. Location
   4. Access to facilities (tea/coffee/toilets)
   5. Support and understanding
8. Was there anything that could have been in place to make it easier for you to
   1. Attend?
   2. Participate?
   3. If you were attending again in the future, would you want anything to be different?
   4. If you were designing your own arts therapies/counselling groups, what would you do?
   5. Where would your ideal location for a group be? What would the room be like?
9. Was there anything related to the COVID-19 pandemic that affected your ability to attend or participate in the groups?
   1. Self-isolation/shielding
   2. Wearing PPE in sessions
   3. Being with others in room
10. Have you participated in any other therapies or groups since the ERA groups?
    1. Was this experience different to the ERA groups?
11. Do you have something else you want to say about your long-term conditions?
12. Do you have other experiences that we haven’t covered that you want to tell us?

General prompts

| Could you tell me a bit more about that please? | What do you mean when you say XX? |
| --- | --- |
| How did you feel about that? | What caused you to…? |
| How did you experience that? | Can you give me some more details? |
