# Supplementary material for: Fighting for care: how can we better support people with multiple long-term conditions who are accessing community mental health groups? A qualitative interview study within a UK arts therapies trial
Source: BMJ Open. 2026 Mar 3;16(3):e103035. doi: 10.1136/bmjopen-2025-103035 (PMC12959026; doi:10.1136/bmjopen-2025-103035)
Supplement: Supplementary file 2 [file bmjopen-2025-103035.reviewer_comments.pdf]

PEER REVIEW HISTORY

BMJ Open publishes all reviews undertaken for accepted manuscripts. Reviewers are asked to complete a checklist review form and are provided with free text boxes to elaborate on their assessment. These free text comments are reproduced below.

ARTICLE DETAILS

Title (Provisional)

Fighting for care: How can we better support people with multiple long-term conditions who are accessing community mental health groups? A qualitative interview study within a UK arts therapies trial.

Authors

Hounsell, Lauren; Millard, Emma; Medlicott, Emma; Fry, Emma; Fernandes, Jane; Carr, Catherine

---

VERSION 1 - REVIEW

**Reviewer**	**1**
**Name**	**Philip, Keir**
**Affiliation**	**National Heart and Lung Institute**
**Date**	**24-Jun-2025**
**COI**	**None**

Many thanks for the opportunity to review this paper. It is an important topic and the manuscript describes a useful addition to the literature. I have a few points that I think need addressing:

Please do a thorough proof read as some errors remain.

Abstract –

Include mention that these are people with MLTCs AND a mental health condition as relevant for better characterising the sample.

Some description of the specific types of interventions used in the trial would be useful

Is the ‘Objective section’ missing the word arts-based from the sentence ‘…engagement in NHS community based therapy’

Introduction-

‘osteoarthritis, asthma and cardio-metabolic factors (diabetes, coronary heart disease) [10] Current clinical guidelines do…’ missing full stop.

Sentence ‘Current clinical guidelines do not reflect the experiences and needs of people with MLTCs [3],’ at lines 5 and 6 repeats on lines 17 and 18.

ERA needs writing in full when first used.

Need to define what the cut off used was for the sentence ‘They also were required to score a minimum level of moderate symptom severity on the Brief Symptom Inventory Global Severity Index (BSI) [24] to be eligible to participate in the study.’

There needs more explanation of reflexivity within data interpretation and analysis, relating to the researchers own background, assumptions and role.

Are some/all researchers trained and experienced in qualitative methods? Can this be included in the manuscript?

What did the participants know about who the interviewers were and the purpose of the research?

A more explicit description of the epistemological approach used would be valuable.

The methods state that they used an approach as outlined by Braun and Clarke 2006 with no other references. It should be noted that since 2006 they have developed their approach substantially. Please review to ensure methods are up to date and justified. Please make sure that the study team includes someone with expertise and experience in qualitative research and has gone though the methods and interpretation in depth and can vouch for its quality.

As an NIHR SWAT study, was the PROMETHEUS team involved?

Does the study address any of the PRioRiTy I and PrioRiTy II James Lind Alliance priorities?

I suggest having a conclusions section at the end of the manuscript

I am unclear why the STROBE guideline was used, it is not really appropriate for a qualitative study. More appropriate would the SRQR or COREQ. Please check with the BMJ Open guidelines

---

**Reviewer**	**2**
**Name**	**Dama, Manish**
**Affiliation**	**Trinity College Dublin School of Medicine**
**Date**	**22-Jul-2025**
**COI**	**None**

I would like to thank the authors for writing this qualitative study exploring the main challenges that individuals with multiple-long term conditions (MLTCs) face when accessing arts therapy in England. This is a useful study which will help guide service delivery of integrate care in East England and provide valuable research for future investigations. However, the study may benefit from the following minor revisions:

1) The ascertainment of physical health conditions via self-report is a methodological limitation. Is it possible to perform a chart review of the 20 participants and identify their physical health diseases? After all, participants may not report all of their physical health comorbidities. For example, not a single participant reported to have hypertension, which is one of the most common physical health disorders in the general population.

2) It would be beneficial to provide a histogram to examine the distribution of participants based on the number of physical health conditions (>1 condition, >2 conditions, etc) that they have. Furthermore, it would be helpful to the reader to get a sense of what the primary mental health disorder was among participants in the sample. It may be useful to classify the number of participants in the 3 groups reported in Methods: 1) F2 (schizophrenia, schizotypal, and delusional disorders); 2) F3 (mood disorders) and 3) F4 (neurotic, stress related and somatoform disorders). This will help the reader get a better sense of the characteristics for the study sample.

3) A key point noted by the authors is that "fatigue, tired, and cognitive disturbances" were reported by participants. If it is possible to perform a medication review, it should be reported in the study. It would be important to see what percentage of participants were on medications that can precipitate these effects. Such medications can include sedating analgesics (e.g., opioids), central nervous system depressants (e.g., benzodiazepines), hypnotics (e.g., z-drugs or antihistamines), and other psychotropics (e.g., atypical antipsychotics or tricyclic antidepressants). The use of these medications may play an important role in driving the formation of Themes 1-3.

4) Is it possible for the authors to compare the factors from Table 1 between the participants in the current study and the study sample from the original randomized controlled trial (ERA trial) to determine if the sample in the current study is representative of the study sample from the ERA trial?

5) The authors also need to comment on how the high prevalence musculoskeletal/rheumatic morbidities among the study sample (nearly half) may have driven the results in the study. This may have played a large role in the development of Themes 1-3 and may have skewed the distribution of participants in the activities that they participated in (e.g., how dance movement therapy was the group with least participation).

6) While not a disorder or disease per se, loneliness and social isolation can play a profound role in health system related factors like accessing care as well as sustaining engagement in the continuity phase of care. This study sample reported these problems by stating: "Spending a lot of time in bed and feeling isolated". It is possible that loneliness and social isolation may have influence the development of Theme 5 (The group was important and valued) as well as Theme 6 (Determination and fighting to get what I need). Therefore, the authors should consider commenting on this.

VERSION 1 - AUTHOR RESPONSE

**Reviewer: 1**<br>**Dr. Keir Philip, National Heart and Lung Institute**	
Many thanks for the opportunity to review this paper. It is an important topic and the manuscript describes a useful addition to the literature.	Thank you for this positive feedback and helpful suggestions to improve the paper.
Please do a thorough proof read as some errors remain.	We have conducted a thorough proof read to correct errors.
Abstract –<br>a)Include mention that these are people with MLTCs AND a mental health condition as relevant for better characterising the sample.<br>b)Some description of the specific types of interventions used in the trial would be useful<br><br>c)Is the 'Objective section' missing the word arts-based from the sentence '…engagement in NHS community based therapy'	a) Sentence rephrased.<br><br>b) We have replaced this with "group arts therapies and group counselling"<br>Abstract, Page 1-2.<br><br>The therapies were arts-based for the arts therapies (art therapy, dance movement therapy and music therapy) and talking based for the active control (group counselling). Participants in this SWAT came from both arms of the trial so we have left the sentence as it is.
Introduction-<br>'osteoarthritis, asthma and cardio-metabolic factors (diabetes, coronary heart disease) [10] Current clinical guidelines do…' missing full stop.<br><br>Sentence 'Current clinical guidelines do not reflect the experiences and needs of people with MLTCs [3],' at lines 5 and 6 repeats on lines 17 and 18.<br><br>ERA needs writing in full when first used.<br><br>Need to define what the cut off used was for the sentence 'They also were required to score a minimum level of moderate symptom severity on the Brief Symptom Inventory Global Severity Index (BSI) [24] to be eligible to participate in the study.'	Full stop now included.<br><br>Duplicate sentence has been removed and the sentence re-ordered to make better sense in the paragraph.<br><br>The full name of the study has been included. Introduction, Page 4, final paragraph.<br><br>The cut-off score of 1.65 has been included. Study population and setting, Page 5, final paragraph.
There needs more explanation of reflexivity within data interpretation and analysis, relating to the researchers own background, assumptions and role.<br>Are some/all researchers trained and experienced in qualitative methods? Can this be included in the manuscript?	We have elaborated on this within our data analysis section, incorporating our contextual statement section and included further details regarding the researchers' backgrounds and experience in qualitative methods.<br><br>Data analysis, Page 8-10.

What did the participants know about who the interviewers were and the purpose of the research?	We have included further information.<br>Study population and setting, Page 6.<br>"Potentially eligible participants", "At all contacts…."<br>Data analysis, Page 9 (last paragraph)-Page 10.
A more explicit description of the epistemological approach used would be valuable.	We have included a few sentences on this at the beginning of our study design.<br>Study design, Page 5: "Our epistemological stance…"
The methods state that they used an approach as outlined by Braun and Clarke 2006 with no other references. It should be noted that since 2006 they have developed their approach substantially. Please review to ensure methods are up to date and justified. Please make sure that the study team includes someone with expertise and experience in qualitative research and has gone through the methods and interpretation in depth and can vouch for its quality.	We have revisited Braun and Clarke's more recent writing (2022), alongside related texts to better elaborate upon, update and substantiate our methods. As a result, and in line with Braun and Clarke's conceptualisation in the context of reflexive thematic analysis, we have better specified our analytic method as 'codebook analysis' given the involvement of a large study team and multiple rounds to ensure coding consistency and coverage. As above, we have outlined further detail about the qualitative experience held by the team. We also consulted with a colleague who holds extensive, primarily qualitative research experience in MLTC populations to ensure quality and we have acknowledged her contributions in the acknowledgements section..
As an NIHR SWAT study, was the PROMETHEUS team involved?	No. This study was funded separately through a specified call from the NIHR NETSCC to further understanding of multimorbidity. Studies within a trial were specifically encouraged in this call. This information can be found in the funding acknowledgement.
Does the study address any of the PRioRiTy I and PrioRiTy II James Lind Alliance priorities?	While this study does not directly address clinical trial recruitment and retention, there are implications that can be drawn from the data. We have highlighted some of these as an addition in the discussion.<br>Discussion, Page 30, "Similarly, within randomised controlled trials…"
I suggest having a conclusions section at the end of the manuscript	We have included a brief conclusion to bring the manuscript to a close. Page 33.
I am unclear why the STROBE guideline was used, it is not really appropriate for a qualitative study. More appropriate would the SRQR or COREQ. Please check with<br>the BMJ Open guidelines	This guideline was used in error. We have now included the SRQR as per journal policy.
Reviewer: 2<br>Dr. Manish H. Dama, Trinity College Dublin School of Medicine	

I would like to thank the authors for writing this qualitative study exploring the main challenges that individuals with multiple-long term conditions (MLTCs) face when accessing arts therapy in England. This is a useful study which will help guide service delivery of integrate care in East England and provide valuable research for future investigations.	Thank you for these positive comments.
1) The ascertainment of physical health conditions via self-report is a methodological limitation. Is it possible to perform a chart review of the 20 participants and identify their physical health diseases? After all, participants may not report all of their physical health comorbidities. For example, not a single participant reported to have hypertension, which is one of the most common physical health disorders in the general population.	This is a limitation of our study. We originally did not have ethical approval to extract this from participant medical records in the trial. While we considered submitting a minor amendment to allow for this, the time elapsed for participants since completing these interviews means that their health statuses may have also changed.<br><br>As per your suggestion 3) below, we did collect medication data at our post-intervention timepoint. We have summarised this to provide a better context to the types of medication participants used which mean that we can infer some wider points about the disorders being treated.<br><br>Having examined this data, we feel fairly confident that most MLTCs were reported accurately, bar that of hypertension, which was not reported at all. We surmise that this may have been through absence of obvious symptoms, or overshadowing of wider health concerns. We have provided a brief comment on this in the discussion.<br>Table 2, Page 12.<br>Discussion, Page 31, final paragraph.
2) It would be beneficial to provide a histogram to examine the distribution of participants based on the number of physical health conditions (>1 condition, >2 conditions, etc) that they have.<br><br><br>Furthermore, it would be helpful to the reader to get a sense of what the primary mental health disorder was among participants in the sample. It may be useful to classify the number of participants in the 3 groups reported in Methods: 1) F2 (schizophrenia, schizotypal, and delusional disorders); 2) F3 (mood disorders) and 3) F4 (neurotic, stress related and somatoform disorders). This will help the	Thank you for this suggestion. Having consulted with our trial statisticians, and compared with cumulative presentations of the data, we feel that the bar chart in its current format is the clearest way of representing the distribution of participants' number of health conditions.<br><br>Thank you for this suggestion, we have included primary mental health diagnosis data in Table 1.<br><br>Table 1, Page 11

reader get a better sense of the characteristics for the study sample.	
3) A key point noted by the authors is that "fatigue, tired, and cognitive disturbances" were reported by participants. If it is possible to perform a medication review, it should be reported in the study. It would be important to see what percentage of participants were on medications that can precipitate these effects. Such medications can include sedating analgesics (e.g., opioids), central nervous system depressants (e.g., benzodiazepines), hypnotics (e.g., z-drugs or antihistamines), and other psychotropics (e.g., atypical antipsychotics or tricyclic antidepressants). The use of these medications may play an important role in driving the formation of Themes 1-3.	Thank you for this suggestion. We have included this information in a further table (Table 2, page 12).
4) Is it possible for the authors to compare the factors from Table 1 between the participants in the current study and the study sample from the original randomized controlled trial (ERA trial) to determine if the sample in the current study is representative of the study sample from the ERA trial?	We have included comparative information about the overall study sample in an additional column in table 1 (page 11).
5) The authors also need to comment on how the high prevalence of musculoskeletal/rheumatic morbidities among the study sample (nearly half) may have driven the results in the study. This may have played a large role in the development of Themes 1-3 and may have skewed the distribution of participants in the activities that they participated in (e.g., how dance movement therapy was the group with least participation).	We have included a commentary and reflection on this in the discussion.<br><br>Discussion, page 31, 4th paragraph "Our participants predominantly…"
6) While not a disorder or disease per se, loneliness and social isolation can play a profound role in health system related factors like accessing care as well as sustaining engagement in the continuity phase of care. This study sample reported these problems by stating: "Spending a lot of time in bed and feeling isolated". It is possible that loneliness and social isolation may have influence the development of Theme 5 (The group was important and valued) as well as Theme 6 (Determination and fighting to get what I need). Therefore, the authors should consider commenting on this.	We have included a commentary and reflection on this in the discussion.<br><br>Discussion, page 31, 4th paragraph "Similarly, while loneliness and social isolation…"

VERSION 2 - REVIEW

**Reviewer**	**2**
**Name**	**Dama, Manish**
**Affiliation**	**Trinity College Dublin School of Medicine**
**Date**	**24-Dec-2025**
**COI**	

The authors have adequately addressed my comments.
